# The effect of ambient PM_2.5_ exposure on survival of lung cancer patients after lobectomy

**DOI:** 10.1186/s12940-023-00976-x

**Published:** 2023-03-07

**Authors:** Changpeng Liu, Dongjian Yang, Yuxi Liu, Heng Piao, Tao Zhang, Xi Li, Erjiang Zhao, Di Zhang, Yan Zheng, Xiance Tang

**Affiliations:** 1 Office for DRGs (Diagnosis Related Groups), Affiliated Cancer Hospital of Zhengzhou University, Henan Cancer Hospital, PO Box 0061, No. 127 Dongming Rd, 450008 Zhengzhou, China; 2 International Peace Maternity and Child Health Hospital, School of Medicine, Shanghai Jiao Tong University, Shanghai, China; 3 Department of Epidemiology, School of Public Health, Fudan University, Shanghai, China

**Keywords:** PM_2.5_, Lung cancer, Lobectomy, Survival

## Abstract

Exposure to fine particulate matter (PM_2.5_) is linked to lung cancer incidence and mortality. However, the impact of PM_2.5_ exposure on lung cancer patients after lobectomy, which remains the primary treatment for early-stage lung cancer, is unknown. Therefore, we investigated the correlation between PM_2.5_ exposure and the survival of lung cancer patients after lobectomy. This study included 3,327 patients with lung cancer who underwent lobectomy procedures. We converted residential addresses into coordinates and estimated individual patients' daily PM_2.5_ and O_3_ exposure levels. A Cox multivariate regression model was used to analyze the specific monthly association between PM_2.5_ exposure and lung cancer survival. Every 10 μg/m^3^ increase in monthly PM_2.5_ concentration in the first and second months after lobectomy increased the risk of death (hazard ratio [HR]: 1.043, 95% confidence interval [CI]: 1.019–1.067 and HR: 1.036, 95% CI: 1.013–1.060, respectively). Non-smokers, younger patients, and patients with longer hospitalization durations had worse survival rates when exposed to greater concentrations of PM_2.5_. High postoperative PM_2.5_ exposure immediately after lobectomy reduced the survival of patients with lung cancer. Patients living in areas with high PM_2.5_ should be offered the opportunity to transfer to areas with better air quality after undergoing lobectomies, to prolong their survival times.

**Supplementary Information:**

The online version contains supplementary material available at 10.1186/s12940-023-00976-x.

## Introduction

Lung cancer is, according to the International Agency for Research on Cancer Global Observatory, now the second most frequently diagnosed cancer globally. The global estimate of new cases of lung cancer has increased to 2.2 million for 2020 (11.4%) [1]. However, lung cancer remains the leading cause of death, with estimated new deaths of 1.8 million (18.0%) [1]. Operative excision continues to be the main curative treatment for early-stage lung cancer because it has the benefits of retaining better postsurgical lung function and lower fatality rates [2].

Air pollution is defined as pollutants in both environmental and domestic air [3]. Environmental air pollution mainly comes from vehicles and industrial and household fuels, and domestic air pollution mainly comes from heating biomass and coal fuels [4]. Fine particulate matter (PM_2.5_) is the most extensively investigated air pollutant and is increasingly used to indicate pollution, with annual average global concentrations ranging from less than 10 μg/m^3^ to more than 100 μg/m^3^ [5]. In 2016, a report using data from the World Health Organization (WHO) estimated that about 4.2 million people were exposed to air pollution to degrees that resulted in reduced life expectancies, mainly due to PM_2.5_. [6]. As the pace of economic growth, urbanization, and industrialization increases, more people are exposed to higher concentrations of PM_2.5_ [7]. Lung cancer is among the most common diseases related to PM_2.5_ [8].

Several studies have evaluated the relationship between PM_2.5_ and the ensuing risk of lung cancer occurrence and fatality. Their findings have indicated that PM_2.5_ may be a risk factor for lung cancer [9–11]. A well-known study based on prospective cohort data gathered by the American Cancer Society [12] declared that prolonged exposure to PM_2.5_ significantly affected survival, with each 10 μg/m^3^ increase being associated with an approximately 8% increase in the risk of death from lung cancer. It is estimated by the Global Burden of Disease (GDB) that 265,000 lung cancer deaths were caused by outdoor air pollution in 2017, which accounted for 14% of all lung cancer deaths [13]. More importantly, particulate matter pollution burden in GDB 2019 increased by 44·6% when compared with GBD 2017 [7]. In China, particularly in some large cities, increased mortality from lung cancer has been noticed in recent years, despite improving medical conditions and a decreasing number of smokers during the same period [14]. Research on the association between lung cancer and PM_2.5_ has received growing attention as air quality has declined. Moreover, Yang et al. reported that long-term exposure to fine particulate air pollution is an important risk factor for lung cancer in China [15].

However, studies on the relationship between PM_2.5_ and lung cancer have mostly centered on the consequent risks of lung cancer morbidity and mortality at the population level, indicating that PM_2.5_ could be a potential contributor to lung cancer [16]. To our knowledge, no prior study has examined the association between ambient PM_2.5_ air pollution and the survival of lung cancer patients following surgical treatment for the condition. Assessing the effect of PM_2.5_ on the survival of lung cancer patients can not only improve the care provided to patients, but also offer a theoretical rationale for public health strategies related to PM_2.5_-induced health implications. Therefore, in this study, we assessed the effect of PM_2.5_ on the survival of lung cancer patients after anatomical lung resection.

## Materials and methods

### Study population

This prospective study was conducted at the Henan Cancer Hospital (HCH), affiliated with the Zhengzhou University. Ethics approval for the study was acquired from the Research and Innovation Department of the hospital, and classified as a service evaluation not requiring review by the HCH Research Ethics Committee. All patients provided signed informed consent before follow-up.

In this prospective study, we selected lung cancer patients who underwent surgery between January 1, 2016, and June 30, 2020. All participants enrolled in the study met the following criteria: 1) the patient’s diagnosis was lung cancer (C33–C34), according to the International Classification of Diseases version 10; and 2) the patient underwent lobectomy. We eliminated patients with concurrent malignant disease or other prior primary cancers. Patients with no current address and patients who had undergone procedures other than lobectomy, such as chest wall resection, pneumonectomy, and segmentectomy, were also excluded. We obtained information on the patients’ socioeconomic statuses, clinical treatments, and follow-up statuses using the electronic medical record systems.

### Cohort follow-up

Patient follow-up was done every three months for the first two years following lobectomy, then every six months until five years post-surgery, and annually thereafter. Each patient's status (dead or alive), date of death (if applicable), and date of last follow-up visit were collected by telephone during the follow-up period.

### PM_2.5_ exposure assessment

The PM_2.5_ [17] and ozone (O_3_) [18] data in this study were obtained from the near real-time Tracking Air Pollution in China (TAP) in China. The TAP database uses a two-level machine learning model combined with a small number of oversampling techniques and a tree-based gap-filling method, and is based on information from multiple data sources. The PM_2.5_ and O_3_ levels were estimated at a 10-km spatial resolution. The data used included PM_2.5_ and O_3_ level observation data, satellite remote sensing aerosol optical depth data, the results of a community multiscale air quality simulation, meteorological reanalysis data, land use data, altitude data, and population data. The model’s average out-of-bag cross-validation R^2^ for the different years was 0.83. We converted residential addresses into coordinates to estimate individual patients' daily PM_2.5_ and O3 exposure levels.

### Statistical methods

We evaluated the atmospheric PM_2.5_ and O_3_ concentrations in specific months from 1–6 months after surgery to analyze the impact of air pollution on postoperative survival, and identify the sensitive period. The distributions of PM_2.5_ and O_3_ after surgery were represented by density plots, means, and quartiles.

A multivariate Cox regression model was used to analyze the specific monthly association between PM_2.5_ exposure and lung cancer survival. We used sex (male or female), age (< 60 years or ≥ 60 years), marital status (married or other), occupation (mainly mental labor, mainly manual labor, and both mental and manual labor), length of hospitalization (≤ 20 days or > 20 days), and medical insurance type (medical insurance for urban residents, new rural cooperative medical insurance, paid out-of-pocket, and other medical insurance) as covariates to adjust the Cox regression models, and added factors such as operation season, smoking (yes or no), and alcohol consumption (yes or no) to subsequent analyses. We also divided patients into high- and low-exposure groups based on whether their PM_2.5_ exposures in the first and second months after surgery were greater or less than the median exposure. The Kaplan–Meier method was then used to draw survival curves, and the log-rank test was used to detect statistical differences in survival between the groups.

We performed a stratified Cox regression analysis based on age, length of hospitalization, sex, and smoking status. The average PM_2.5_ exposure concentrations at one month, 1–3 months, and 1–6 months after surgery were also evaluated to analyze the long-term effects of PM_2.5_ exposure. In our sensitivity analysis, we adjusted the O_3_ exposure concentration for a specific month in the Cox regression model to exclude the potential effects of other air pollutants. We adjusted for different age groups and chronic diseases (hypertension, diabetes, cardiovascular disease) in our sensitivity analysis. To exclude the effect of preoperative air pollution exposure, we also adjusted for PM_2.5_ exposure and O_3_ exposure in the model for six months prior to the operations.

All analyses were performed using R version: 4.0.1 (https://www.r-project.org/), and bilateral *P* values of < 0.05 were considered statistically significant.

## Results

There were 3,327 participants in our study. The mean follow-up period was 2.14 ± 1.25 years. Of the participants, 58.60% were men, 53.28% were older than 60 years, 53.83% were non-smokers, 26.91% had smoked in the past but had quit, 27.23% did not drink alcohol, and 27.07% had drunk alcohol in the past but had quit. Approximately half (51.04%) of hospitalization stays were longer than 20 days. The most common season for lung cancer surgery was the summer (29.28%) (Table 1).

**Table 1:** Characteristics of participants in postoperative patients with lung cancer

Characteristics	Lung cancer patients [N (%)]
Gender	
Male	1829(58.60)
Female	1292(41.40)
Ethnicity	
Han	3099(99.30)
Others	22(0.70)
Marital status	
Married	3058(97.98)
Others	63(2.02)
Occupation type	
Mainly mental labor	297(9.52)
Mainly manual labor	1954(62.61)
Both mental and manual labor	870(27.88)
Medical insurance type	
Medical insurance for urban residents	747(23.93)
New rural cooperative medical insurance	1590(50.95)
At one's own expense	610(19.55)
Other medical insurance	174(5.58)
Hospitalization days	
≤ 20 days	1528(48.96)
> 20 days	1593(51.04)
Age	
≤ 50	439(14.07)
50 to ≤ 60	1019(32.65)
60 to ≤ 70	1242(39.79)
> 70	421(13.49)
Drinking	
No	1786(57.23)
Have drunk but stopped drinking	845(27.07)
Currently drinking	490(15.7)
Smoking	
No	1680(53.83)
Have smoked but stopped smoking	840(26.91)
Currently smoking	601(19.26)
Operation season	
Spring	641(23.23)
Summer	817(29.61)
Autumn	675(24.47)
Winter	626(22.69)
Hypertension	
No	2435(78.02)
Yes	686(21.98)
Diabetes	
No	2815(90.20)
Yes	306(9.80)
Cardiovascular disease	
No	2920(93.56)
Yes	201(6.44)

**Fig. 1:**
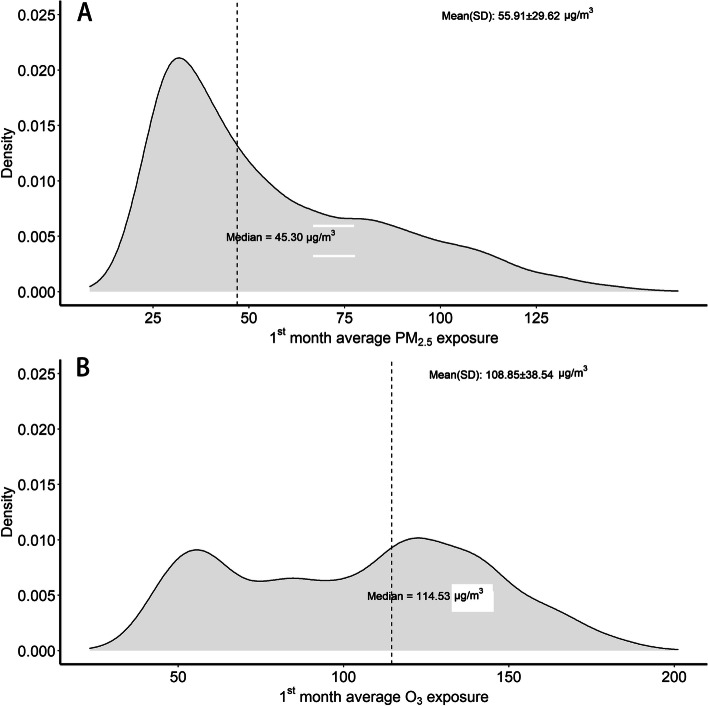
The distribution of mean monthly PM_2.5_ exposures (**A**) and O_3_ exposures (**B**) during the first month after lung cancer surgery. PM_2.5_: fine particulate matter; O_3_: ozone

During the first month after lung cancer surgery, the average specific monthly PM_2.5_ exposure concentration was 55.91 ± 29.62 μg/m^3^, and the average monthly O_3_ exposure concentration was 108.85 ± 38.54 mg/m^3^ (Fig. 1 and Table S1). During the study period the median (interquartile range) monthly environmental-specific PM_2.5_ and O_3_ exposures were 45.30 μg/m^3^ (32.63–73.20 μg/m^3^) and 114.53 mg/m^3^ (72.43–138.17 mg/m^3^), respectively.

### The association between specific monthly PM_2.5_ exposure and survival of lung cancer patients after surgery

**Fig. 2:**
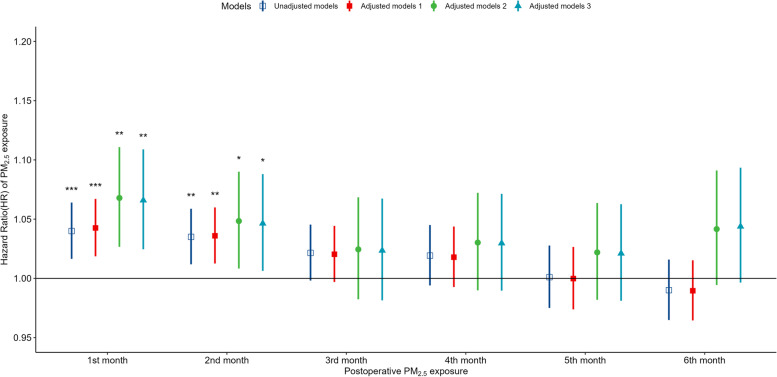
HRs of postoperative death in lung cancer patients in association with specific monthly (1–6 months after surgery) exposure to PM_2.5_. Model 1 was adjusted for age at operation, sex, occupation type, ethnicity, marital status, and length of hospitalization. Model 2 was adjusted for the factors in Model 1, as well as operation season. Model 3 was adjusted for the factors in Model 2, as well as for smoking and alcohol consumption. HRs: hazard ratios; PM_2.5_: fine particulate matter

**Fig. 3:**
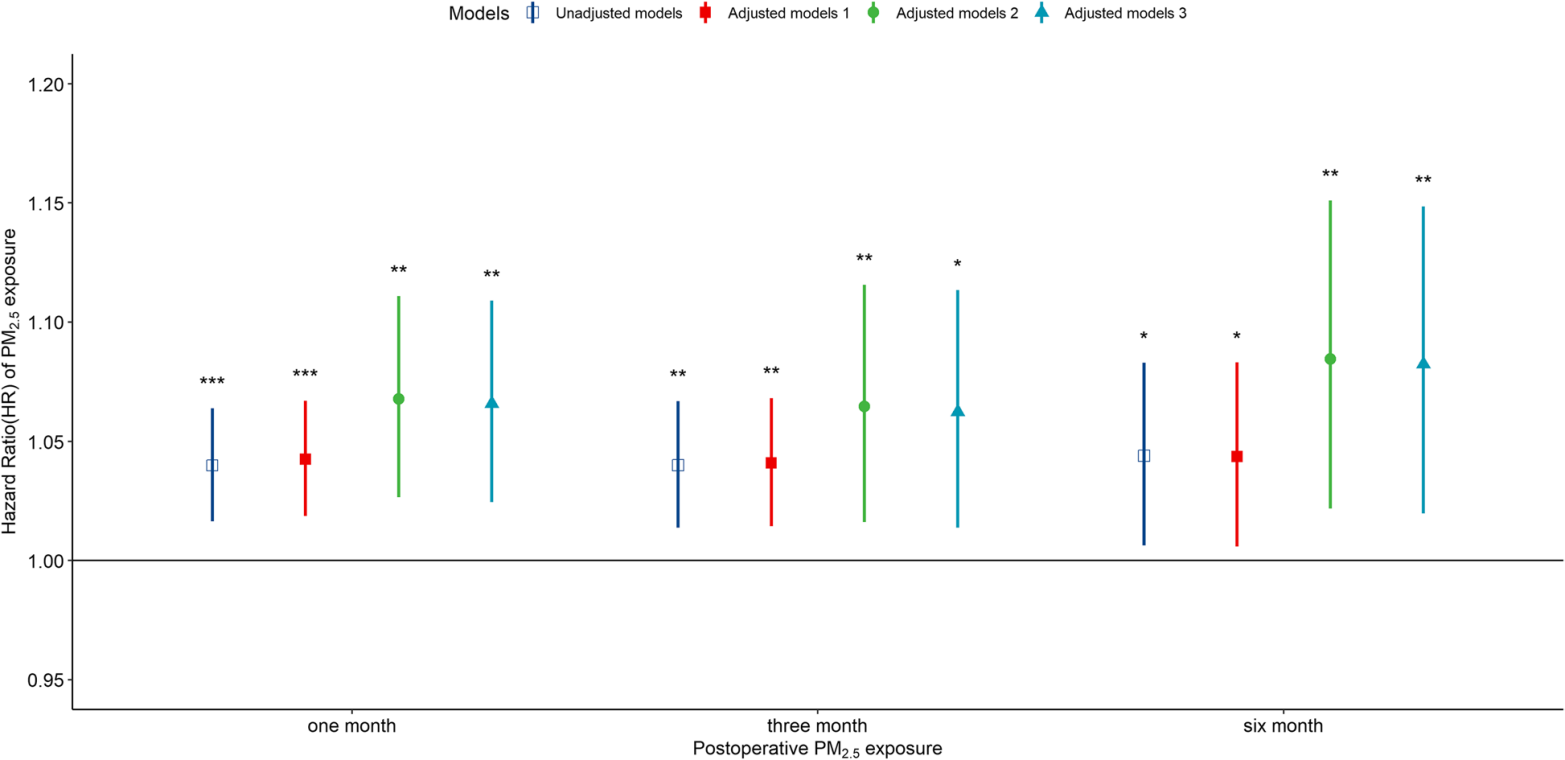
HRs of postoperative death in lung cancer patients in association with long-term exposure (1 month: 1 month, 3 months: 1–3 months, 6 months: 1–6 months) to PM_2.5_. The model was adjusted for age at operation, sex, occupation type, ethnicity, marital status, length of hospitalization, smoking, and hospitalization days, smoking, and drinking. HRs: hazard ratios; PM_2.5_: fine particulate matter

The average specific monthly residential PM_2.5_ exposures were used to analyze the effect of PM_2.5_ exposure on the survival of lung cancer patients after surgery. As shown in Fig. 2, every 10 μg/m^3^ increase in monthly PM_2.5_ concentration in the first and second months after surgery increased the risk of death (hazard ratio [HR]: 1.043, 95% confidence interval [CI]: 1.019–1.067 and HR: 1.036, 95% CI: 1.013–1.060, respectively). PM_2.5_ concentration was still a significant independent predictor after adjusting for season during which the surgery took place, smoking, and alcohol consumption. The PM_2.5_ concentrations within 1, 3, and 6 months after surgery were averaged to evaluate the long-term exposure effect. As shown in Fig. 3, for every increase of 10 μg/m^3^ in the average PM_2.5_ exposure within 1, 3, and 6 months after surgery, the risk of death increased significantly (1 month, HR: 1.043, 95% CI: 1.019–1.067; 3 months, HR: 1.048, 95% CI: 1.014–1.068; and 6 months, HR: 1.044, 95% CI: 1.006–1.083).

**Fig. 4:**
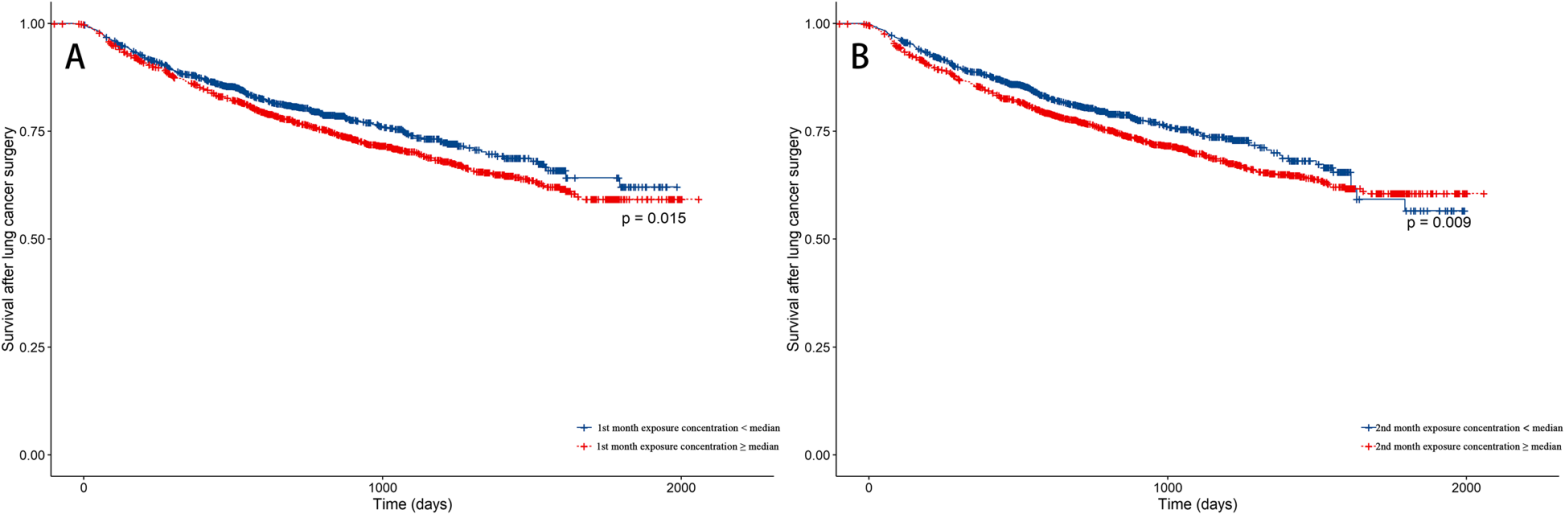
Survival after lung cancer surgery in patients with exposure to different PM_2.5_ concentrations. The high-exposure group was defined as those exposed to a PM_2.5_ concentration above the median, and the low-exposure group was defined as those exposed to a PM_2.5_ concentration below the median. PM_2.5_: fine particulate matter

Patients were divided into high- and low-exposure groups based on the average specific monthly PM_2.5_ concentrations, and survivals in the first and second months after surgery were analyzed. As shown in Fig. 4, the survivals in the first and second months after surgery were significantly lower in the high-exposure group than in the low-exposure group (*P* = 0.015 and 0.009, respectively).

### Stratification analysis

**Fig. 5:**
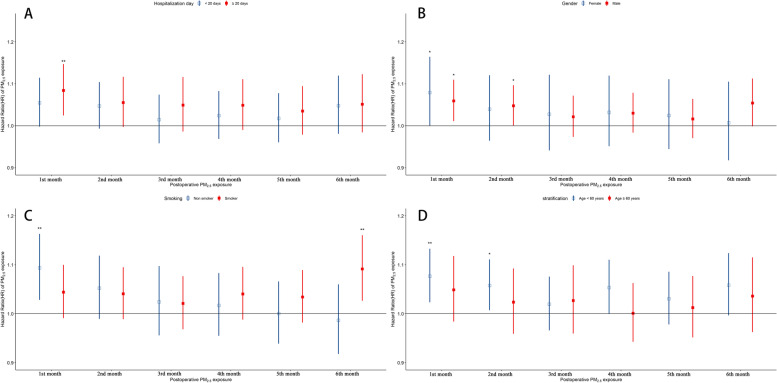
HRs of postoperative death in lung cancer patients in relation to specific monthly (1–6 months after operation) exposure to PM_2.5_ stratified by length of hospitalization (A), sex (B), smoking (C), and age at operation (D). Model 1 was adjusted for age at operation, sex, occupation type, ethnicity, marital status, and length of hospitalization. Model 2 was adjusted for the factors in Model 1, as well as operation season. Model 3 was adjusted for the factors in Model 2, as well as smoking and alcohol consumption. Stratification factors were not adjusted. HRs: hazard ratios; PM_2.5_: fine particulate matter

We stratified the patients according to the length of hospitalization (< 20 days or ≥ 20 days), sex (male or female), smoking (yes or no), and age at operation (< 60 years or ≥ 60 years) and assessed the association between PM_2.5_ exposure and survival. As shown in Fig. 5A, lung cancer patients with longer hospitalization stays had higher risks of death (HR: 1.084, 95% CI: 1.024–1.147) for every 10 μg/m^3^ increase in PM_2.5_ concentration in the first month after surgery, but there was no statistical association between PM_2.5_ concentration and mortality in the second month after surgery. Non-smoking lung cancer patients had higher risks of death (HR: 1.094, 95% CI: 1.028–1.163) for every 10 μg/m^3^ increase in PM_2.5_ concentration in the first month after surgery (Fig. 5C), and lung cancer patients younger than 60 years had higher risks of death (first month, HR: 1.094, 95% CI: 1.028–1.163; second month, HR: 1.058, 95% CI: 1.007–1.111) for every 10 μg/m^3^ increase in PM_2.5_ concentration in the first and second months after surgery (Fig. 5D).

### Sensitivity analysis

**Fig. 6:**
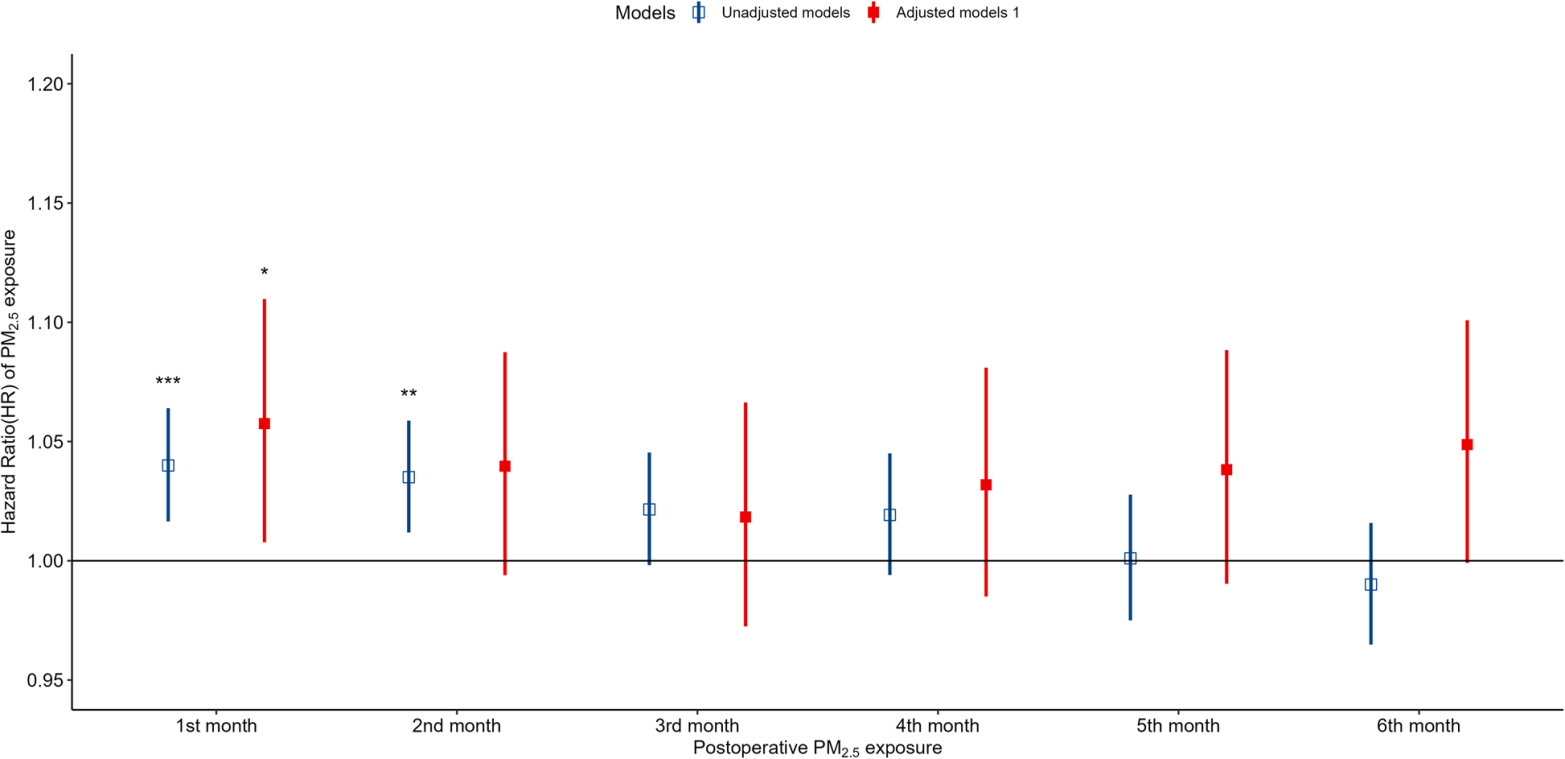
HRs of postoperative death in lung cancer patients in association with specific monthly (1–6 months after operation) exposure to PM_2.5_ adjusted for specific monthly O_3_ exposure. The model was adjusted for age at operation, sex, occupation type, ethnicity, marital status, length of hospitalization, smoking, and alcohol consumption. HRs: hazard ratios; PM_2.5_: fine particulate matter; O_3_: ozone

Specific monthly O_3_ exposure was added to the Cox regression model to adjust for the effects of other air pollutants. As shown in Fig. 6, in the first month after surgery, for every 10 μg/m^3^ increase in PM_2.5_ concentration, the risk of death in lung cancer patients still increased significantly, but the effect at the second month after surgery was not statistically significant. The potential effects of PM_2.5_ exposure and O_3_ exposure in the six months prior to surgery were also adjusted in the model. The association between PM_2.5_ and postoperative survival remained significant in the first and second postoperative months after adjusting for preoperative 6-month PM_2.5_ exposure, O_3_ exposure, and both (Figure S1). The association between first- and second-month postoperative exposure and postoperative survival remained robust after adjusting for different age subgroups and chronic disease in the model (Figure S2 and Figure S3).

## Discussion

One of the main goals of this study was to determine the association between PM_2.5_ exposure and survival of lung cancer patients after surgery. Although not all our results were significant, the overall trend suggested that high postoperative levels of PM_2.5_ reduced the survival of lung cancer patients after surgical lobectomies. Our data suggest that for every 10 μg/m^3^ additional monthly PM_2.5_ exposure in the first and second postoperative months, the risk of death increases (HR: 1.043, 95% CI: 1.019–1.067 and HR: 1.036, 95% CI: 1.013–1.060, respectively). This may be true not only in patients treated surgically, as Fu et al. also identified a positive association between PM_2.5_ exposure and lung cancer mortality rates, including patients receiving other treatment modalities [19]. They found an overall trend that provinces in China with higher PM_2.5_ levels had higher mortality rates among lung cancer patients.

Patients’ demographic characteristics that may have also affected survival (sex, age, marital status, occupation, length of hospitalization, and medical insurance type) were controlled for in our multivariate analyses. To adjust for the effects of other air pollutants, specific monthly O_3_ exposure was also integrated into our Cox regression model in the sensitivity analysis. The PM_2.5_ and O_3_ data used in this study came from the near-real-time TAP in China. We used the Baidu Maps API to convert residential addresses into coordinates and estimate the daily PM_2.5_ and O_3_ exposure. We were therefore able to accurately assess the exposure of patients at the individual level. However, there are also several key limitations to our study. First, data on tumor-node-metastasis stage and pathological classification, which are important for forecasting the survival of patients with lung carcinoma [20], were unavailable. Therefore, we used the length of hospitalization to approximate disease severity. Second, the influence of indoor air pollution was not taken into consideration because of a lack of related data. However, a former study showed that a high levels of indoor air pollution may also induce respiratory symptoms and impair lung function [21]. Third, we did not have the data concerning certain comorbidities such as lung function or chronic obstructive pulmonary diseases (COPD) of our cancer patients. However, we conducted a sensitivity analysis that controlled for hypertension, diabetes, and cardiovascular diseases. Fourth, we did not have data regarding long term exposure prior to surgery. However, we adjusted PM_2.5_ exposure for about six months prior to surgery in our sensitivity analyses. Lastly, we did not have information on residential changes, and only used permanent addresses for exposure assessment. As most older individuals tend to have fixed residential addresses, we assumed only a small proportion of our subjects had changed their places of residence during the follow-up period, and thus that this factor did not introduce any substantial bias to our study results.

The causal relationship between PM_2.5_ concentration and carcinogenic risk has been well demonstrated by epidemiological studies. Studies performed in the past decade have mostly focused on the development of exposure–response models to assess lung cancer mortality and morbidity risks in relation to PM_2.5_ exposure. However, to our knowledge, no prior study has examined the relationship between ambient PM_2.5_ exposure and the survival of lung cancer patients after surgery. In this study, we found that increases in PM_2.5_ exposure in the first and second months after surgery increased the risk of mortality. In a previous study in China, there was a 5.2% increase in lung cancer mortality for every 10 μg/m^3^ increase in PM_2.5_ concentration [22]. This is comparable to the results of our study. Even stronger correlations between PM_2.5_ exposure and mortality in lung cancer patients have been reported in North America [23, 24]. The average PM_2.5_ concentration in this study was 55.91 μg/m^3^, which is higher than the levels in most previous studies in Western populations, which had mean concentrations ranging from 6.6 μg/m^3^ to 13.0 μg/m^3^ [24]. The levels of PM_2.5_ may therefore influence the effect of PM_2.5_ on the survival of lung cancer patients. These differences may also be attributable, however, to geographical diversity and patient heterogeneity. PM_2.5_ exposure may affect postoperative survival through potential effects on respiratory function, cancer recurrence, and complications, in patients undergoing lung cancer surgery [25, 26]. In our sensitivity analysis, the risk of death in lung cancer patients remained significantly higher after adjusting for other comorbidities. One study conducted among the general population in a hospital reported that a 10 mg/m^3^ increase in PM_2.5_ may lead to a 1.04% (95% CI 0.52% to 1.56%) increase in mortality rate [27]. The impact of PM_2.5_ exposure on the mortalities of patients with respiratory diseases was larger than that of cardiovascular disease, 1.51% (1.01% to 2.01%) vs 0.84% (0.41% to 1.28%) [27]. We hypothesize that PM_2.5_ affects patient survival primarily in terms of respiratory function and cancer recurrence, but we cannot exclude the potential influences of comorbidities and complications on postoperative survival. However, the specific biological mechanisms by which PM_2.5_ affects postoperative survival in lung cancer patients still need to be further studied. Another noteworthy result of our study was that PM_2.5_ exposure had a significant effect on patients with longer hospital stays. This is likely primarily due to the fact that the efficacy of the immune system decreases as lung cancer disease severity increases [28]. Therefore, patients with longer hospital stays are more vulnerable to the health effects caused by air pollution [29]. Some previous studies have reported that the effect of PM_2.5_ on survival of lung cancer patients was more pronounced among former or current smokers [24]. However, in our study, PM_2.5_ exposure affected the survival of never-smokers, but not that of ever-smokers. This may because smoking produces particulates also contained in PM_2.5_, which are directly absorbed into the body when smoking and negatively affect lung health [30]. Therefore, the effect of PM_2.5_ exposure may have been obscured by smoking.

Our results also showed that there was a relatively stronger correlation between survival and PM_2.5_ concentration in the younger population compared to the older population, which was different from the results of a previous study [30]. This could be because younger patients have higher rates of recovery and more outdoor physical activity; therefore, they are more easily exposed to PM_2.5_ [31].

Several possible mechanisms for the correlation between PM_2.5_ concentration and lung cancer development have been proposed. Under exposure to PM_2.5_, epigenetic and microenvironmental alterations mediated by microRNA dysregulation, DNA methylation, cell autophagy, and apoptosis may activate oncogene-associated pathways to induce carcinomatosis of the lung [32]. Chao et al. found that chronic PM_2.5_ exposure induced lung cancer development by enhancing interleukin-17a (IL- 17a)-regulated proliferation and metastasis, and increased the risk of non-small cell lung cancer progression [33]. In their study, PM_2.5_ exposure resulted in significant lung damage. However, IL-17a-knockout mice displayed significantly less pulmonary impairment after PM_2.5_ exposure. Therefore, PM_2.5_ exposure may reduce survival through IL-17a signaling.

Intermediate actions are required to enhance air quality and minimize the impact of PM_2.5_ on patients [34]. The regulation of PM_2.5_ is an urgent issue for the Chinese government. Our results suggest that China needs to change its economic development pattern to promote air quality. Economic growth should be combined with protection of the environment to build a sustainable economic development pattern. Moreover, patients who undergo pulmonary lobectomy in regions with high PM_2.5_ should be offered the opportunity to transfer to areas with better air quality, to prolong their survival times.

## Conclusion

PM_2.5_ exposure in the first two months after surgery may reduce the survival of patients with lung cancer. The results of this study emphasize the importance of improving air quality. Further studies regarding the possible mechanism by which PM_2.5_ exacerbates negative health effects in lung cancer patients are needed, to confirm the inferences made from the data collected in this survey.

## Supplementary Information

**Additional file 1:** **Table S1.** Distribution of air pollutant specific-monthly exposure after lung cancer surgery. **Table S2.** Distribution of air pollutant exposure after lung cancer surgery. **Figure S1.** Hazard ratios (HRs) of postoperative death among lung cancer patients in association with specific monthly exposure to PM_2.5_, after adjusting for air pollution before the operation. Model 1 was adjusted for age at operation, sex, occupation type, ethnicity, marital status, length of hospitalization, operation season, and PM_2.5_ exposure for 6 months before the operation. Model 2 was adjusted for age at operation, sex, occupation type, ethnicity, marital status, length of hospitalization, operation season, and O_3_ exposure for 6 months before the operation. Model 3 was adjusted for age at operation, sex, occupation type, ethnicity, marital status, length of hospitalization, operation season, O_3_ exposure for 6 months before the operation, and PM_2.5_ exposure for 6 months before the operation. **Figure S2.** Hazard ratios (HRs) of postoperative death among lung cancer patients in association with specific monthly exposure to PM_2.5_, after adjusting for chronic diseases. Model 1 was adjusted for age at operation, sex, occupation type, ethnicity, marital status, length of hospitalization, operation season, and cardiovascular disease. Model 2 was adjusted for age at operation, sex, occupation type, ethnicity, marital status, length of hospitalization, operation season, and hypertension. Model 3 was adjusted for age at operation, sex, occupation type, ethnicity, marital status, length of hospitalization, operation season, and diabetes. **Figure S3.** Hazard ratios (HRs) of postoperative death among lung cancer patients in association with specific monthly exposure to PM_2.5_, after adjusting for different age subgroups. Model 1 was adjusted for age at operation, sex, occupation type, ethnicity, marital status, and length of hospitalization. Model 2 was adjusted for the factors in Model 1 and operation season. Model 3 was adjusted for the factors in Model 2 and smoking and alcohol consumption. Age groups are <50, 50~59, 60~69, and ≥70.

## Data Availability

The datasets generated or analysed in this study are available through Xiance Tang at Henan Cancer Hospital.

## Abbreviations

PM_2.5_: Fine particulate matter
HR: Hazard ratio
CI: Confidence interval
PM: Particulate matter
WHO: World health organization
HCH: Henan cancer hospital
O_3_: Ozone
TAP: Tracking of air pollution
IL-17a: Interleukin 17a
