# Supplementary material for: The effect of ambient PM2.5 exposure on survival of lung cancer patients after lobectomy
Source: Environ Health. 2023 Mar 7;22:23. doi: 10.1186/s12940-023-00976-x (PMC9990243; doi:10.1186/s12940-023-00976-x)
Supplement: Supplementary file 1 — Additional file 1: Table S1. Distribution of air pollutant specific-monthly exposure after lung cancer surgery. Table S2. Distribution of air pollutant exposure after lung cancer surgery. Figure S1. Hazard ratios (HRs) of postoperative death among lung cancer patients in association with specific monthly exposure to PM2.5, after adjusting for air pollution before the operation. Model 1 was adjusted for age at operation, sex, occupation type, ethnicity, marital status, length of hospitalization, operation season, and PM2.5 exposure for 6 months before the operation. Model 2 was adjusted for age at operation, sex, occupation type, ethnicity, marital status, length of hospitalization, operation season, and O3 exposure for 6 months before the operation. Model 3 was adjusted for age at operation, sex, occupation type, ethnicity, marital status, length of hospitalization, operation season, O3 exposure for 6 months before the operation, and PM2.5 exposure for 6 months before the operation. Figure S2. Hazard ratios (HRs) of postoperative death among lung cancer patients in association with specific monthly exposure to PM2.5, after adjusting for chronic diseases. Model 1 was adjusted for age at operation, sex, occupation type, ethnicity, marital status, length of hospitalization, operation season, and cardiovascular disease. Model 2 was adjusted for age at operation, sex, occupation type, ethnicity, marital status, length of hospitalization, operation season, and hypertension. Model 3 was adjusted for age at operation, sex, occupation type, ethnicity, marital status, length of hospitalization, operation season, and diabetes. Figure S3. Hazard ratios (HRs) of postoperative death among lung cancer patients in association with specific monthly exposure to PM2.5, after adjusting for different age subgroups. Model 1 was adjusted for age at operation, sex, occupation type, ethnicity, marital status, and length of hospitalization. Model 2 was adjusted for the factors in Model 1 and [file 12940_2023_976_MOESM1_ESM.docx]

Table S1 Distribution of air pollutant specific-monthly exposure after lung cancer surgery

| Specific-monthly exposure | Pollutant distribution | | | | | | |
| --- | --- | --- | --- | --- | --- | --- | --- |
|  | Percentile distribution | | | | | | Mean ± SD |
|  | 5% | 25% | 50% | 75% | 95% | 100% |  |
| PM_2.5_ exposure (μg/m^3^) |  |  |  |  |  |  |  |
| 1st month | 24.47 | 32.63 | 45.30 | 73.20 | 115.30 | 154.73 | 55.91 ± 29.62 |
| 2nd month | 23.17 | 31.63 | 43.97 | 75.87 | 113.23 | 158.03 | 55.19 ± 29.85 |
| 3rd month | 22.17 | 31.07 | 43.97 | 71.53 | 115.17 | 160.60 | 54.54 ± 30.09 |
| 4th month | 22.90 | 32.77 | 44.83 | 67.63 | 111.13 | 159.33 | 53.66 ± 27.70 |
| 5th month | 24.20 | 33.63 | 46.17 | 68.07 | 109.90 | 144.20 | 54.20 ± 26.89 |
| 6th month | 25.30 | 34.87 | 48.63 | 73.13 | 112.60 | 151.30 | 56.58 ± 27.61 |
| O_3_ exposure (μg/m^3^) |  |  |  |  |  |  |  |
| 1st month | 47.03 | 72.43 | 114.53 | 138.17 | 167.17 | 199.10 | 108.85 ± 38.54 |
| 2nd month | 46.53 | 75.87 | 114.77 | 137.77 | 166.43 | 201.07 | 108.47 ± 38.23 |
| 3rd month | 48.20 | 79.93 | 112.03 | 135.90 | 164.97 | 200.57 | 108.35 ± 36.27 |
| 4th month | 49.47 | 81.17 | 111.60 | 131.63 | 160.37 | 193.13 | 107.06 ± 33.87 |
| 5th month | 48.93 | 80.13 | 108.20 | 130.37 | 158.60 | 190.93 | 105.22 ± 33.81 |
| 6th month | 47.60 | 73.13 | 106.00 | 131.67 | 165.10 | 192.43 | 103.72 ± 36.68 |

**Table S2** Distribution of air pollutant exposure after lung cancer surgery

|  | PM_2.5_ exposure (μg/m^3^) | |  | O_3_ exposure (μg/m^3^) | |
| --- | --- | --- | --- | --- | --- |
| Characteristics | Median (25% to 75% quantile) | Mean ± SD |  | Median (25% to 75% quantile) | Mean ± SD |
| Gender |  |  |  |  |  |
| Male | 38.64(54.09 to 70.83) | 55.69±19.67 |  | 86.14(106.81 to 124.87) | 106.26±22.85 |
| Female | 37.64(51.60 to 68.42) | 54.05±18.84 |  | 87.22(108.31 to 126.50) | 107.91±22.82 |
| Ethnicity |  |  |  |  |  |
| Han | 39.99(55.85 to 69.77) | 57.91±21.25 |  | 86.60(110.71 to 122.25) | 106.11±22.55 |
| Others | 38.06(53.08 to 69.82) | 54.99±19.33 |  | 86.56(107.44 to 125.58) | 106.95±22.85 |
| Marital status |  |  |  |  |  |
| Married | 38.11(53.17 to 69.88) | 55.05±19.38 |  | 86.60(107.43 to 125.45) | 106.91±22.80 |
| Others | 36.63(50.36 to 67.97) | 53.24±17.75 |  | 84.56(110.38 to 127.34) | 108.50±25.01 |
| Occupation type |  |  |  |  |  |
| Mainly mental labor | 42.51(58.89 to 75.89) | 59.56±20.70 |  | 82.17(101.91 to 124.77) | 103.70±25.06 |
| Mainly manual labor | 37.04(51.64 to 67.72) | 53.57±18.70 |  | 87.96(108.63 to 125.31) | 107.67±21.92 |
| Both mental and manual labor | 39.74(54.67 to 71.30) | 56.69±19.92 |  | 84.22(106.81 to 126.42) | 106.43±23.98 |
| Medical insurance type |  |  |  |  |  |
| Medical insurance for urban residents | 40.26(54.08 to 70.94) | 56.41±19.73 |  | 87.06(108.91 to 129.65) | 108.87±24.75 |
| New rural cooperative medical insurance | 36.33(51.31 to 67.70) | 53.33±18.98 |  | 86.65(107.72 to 124.80) | 106.87±21.86 |
| At one's own expense | 41.02(54.83 to 71.90) | 57.06±19.64 |  | 84.88(105.81 to 123.46) | 104.94±22.69 |
| Other medical insurance | 40.02(56.42 to 73.03) | 57.19±18.69 |  | 89.16(102.74 to 126.35) | 106.43±23.16 |
| Hospitalization days |  |  |  |  |  |
| ≤ 20 days | 38.43(53.75 to 70.09) | 55.30±19.35 |  | 85.68(107.12 to 126.73) | 106.89±23.4 |
| > 20 days | 37.79(52.56 to 69.33) | 54.74±19.34 |  | 87.74(107.84 to 124.72) | 107.00±22.31 |
| Age |  |  |  |  |  |
| ≤60 | 37.48(52.23 to 68.91) | 54.47±19.58 |  | 86.07(107.39 to 125.47) | 106.81±22.99 |
| >60 | 39.16(53.77 to 70.39) | 55.49±19.12 |  | 86.76(107.61 to 125.64) | 107.07±22.73 |
| Drinking |  |  |  |  |  |
| No | 38.02(52.66 to 68.84) | 54.60±19.06 |  | 87.35(108.18 to 126.34) | 107.75±22.79 |
| Have drunk but stopped drinking | 38.14(53.09 to 70.36) | 55.16±19.42 |  | 86.03(106.76 to 124.92) | 106.18±22.94 |
| Currently drinking | 38.14(54.97 to 71.43) | 56.26±20.20 |  | 84.69(106.02 to 124.35) | 105.32±22.79 |
| Smoking |  |  |  |  |  |
| No | 37.70(51.98 to 68.53) | 54.20±19.02 |  | 87.61(108.68 to 126.32) | 107.92±22.71 |
| Have smoke but stopped smoking | 39.50(54.02 to 71.01) | 55.76±19.31 |  | 85.44(105.65 to 125.44) | 105.84±23.25 |
| Currently smoking | 38.21(54.54 to 71.37) | 56.23±20.21 |  | 85.83(106.57 to 124.22) | 105.76±22.57 |
| Operation season |  |  |  |  |  |
| Spring | 68.88(76.97 to 87.72) | 77.60±13.41 |  | 73.66(78.51 to 82.57) | 77.87±7.21 |
| Summer | 30.27(34.62 to 40.69) | 36.14±8.36 |  | 125.88(134.24 to 141.35) | 133.42±10.42 |
| Autumn | 44.79(55.30 to 65.55) | 55.86±14.16 |  | 88.58(97.70 to 108.83) | 98.79±13.95 |
| Winter | 49.95(59.01 to 69.50) | 59.08±13.90 |  | 100.8(109.78 to 120.06) | 110.69±12.87 |
| Hypertension |  |  |  |  |  |
| No | 38.38(53.18 to 70.05) | 55.18±19.41 |  | 85.99(107.12 to 125.03) | 106.53±22.86 |
| Yes | 37.70(52.85 to 68.93) | 54.42±19.09 |  | 90.4(109.72 to 127.73) | 108.42±22.75 |
| Diabetes |  |  |  |  |  |
| No | 37.88(53.07 to 69.70) | 54.95±19.39 |  | 86.35(107.44 to 125.71) | 107.02±22.90 |
| Yes | 39.89(53.29 to 70.63) | 55.62±18.88 |  | 87.80(107.54 to 123.40) | 106.24±22.35 |
| Cardiovascular disease |  |  |  |  |  |
| No | 38.33(53.15 to 69.97) | 55.13±19.42 |  | 86.58(107.49 to 125.58) | 106.95±22.85 |
| Yes | 37.04(51.97 to 68.96) | 53.30±18.17 |  | 81.86(109.27 to 121.05) | 105.42±23.20 |


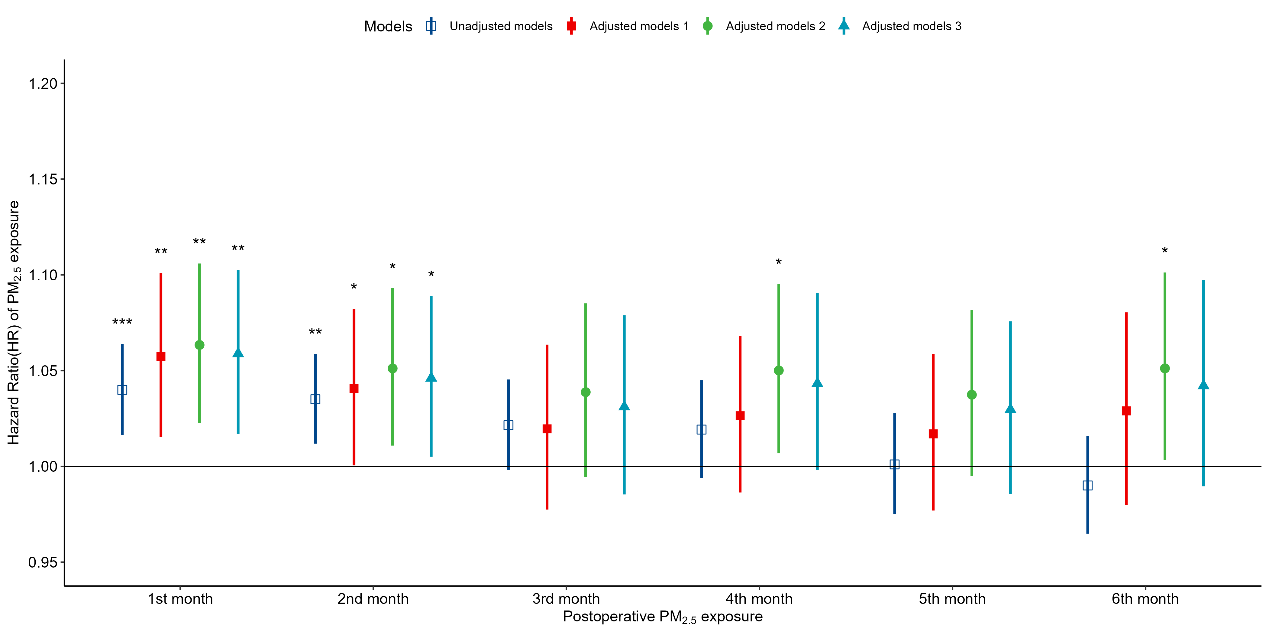


Figure S1 Hazard ratios (HRs) of postoperative death among lung cancer patients in association with specific monthly exposure to PM_2.5_, after adjusting for air pollution before the operation. Model 1 was adjusted for age at operation, sex, occupation type, ethnicity, marital status, length of hospitalization, operation season, and PM_2.5_ exposure for 6 months before the operation. Model 2 was adjusted for age at operation, sex, occupation type, ethnicity, marital status, length of hospitalization, operation season, and O_3_ exposure for 6 months before the operation. Model 3 was adjusted for age at operation, sex, occupation type, ethnicity, marital status, length of hospitalization, operation season, O_3_ exposure for 6 months before the operation, and PM_2.5_ exposure for 6 months before the operation.


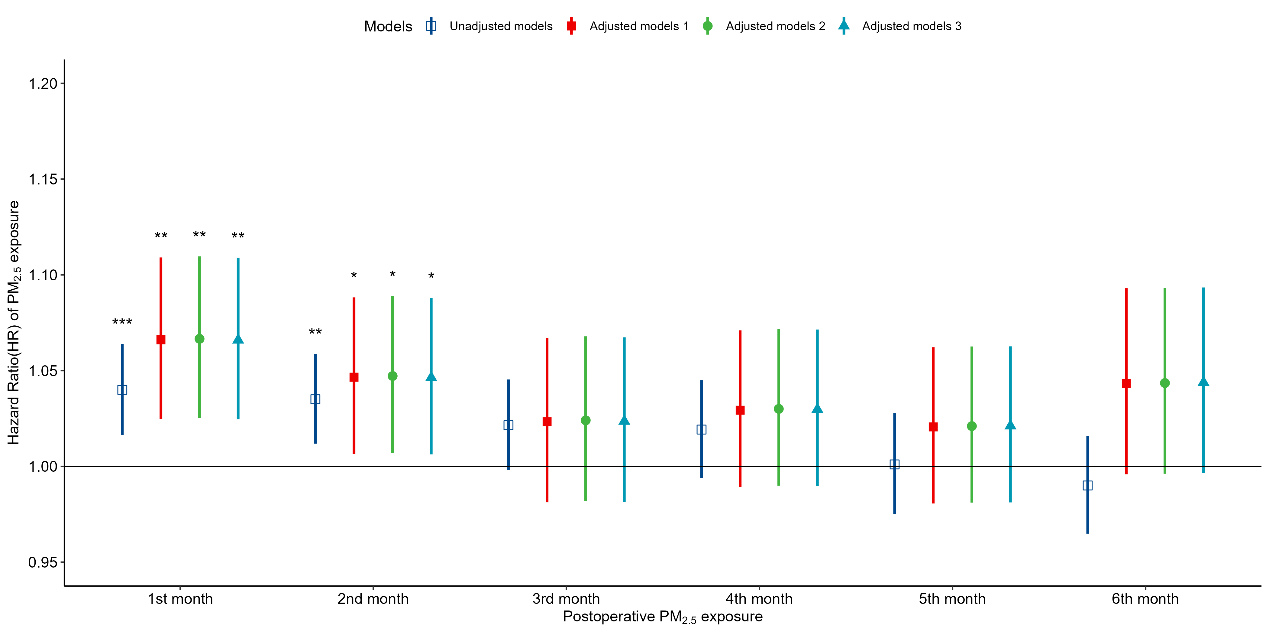


Figure S2 Hazard ratios (HRs) of postoperative death among lung cancer patients in association with specific monthly exposure to PM_2.5_, after adjusting for chronic diseases. Model 1 was adjusted for age at operation, sex, occupation type, ethnicity, marital status, length of hospitalization, operation season, and cardiovascular disease. Model 2 was adjusted for age at operation, sex, occupation type, ethnicity, marital status, length of hospitalization, operation season, and hypertension. Model 3 was adjusted for age at operation, sex, occupation type, ethnicity, marital status, length of hospitalization, operation season, and diabetes.


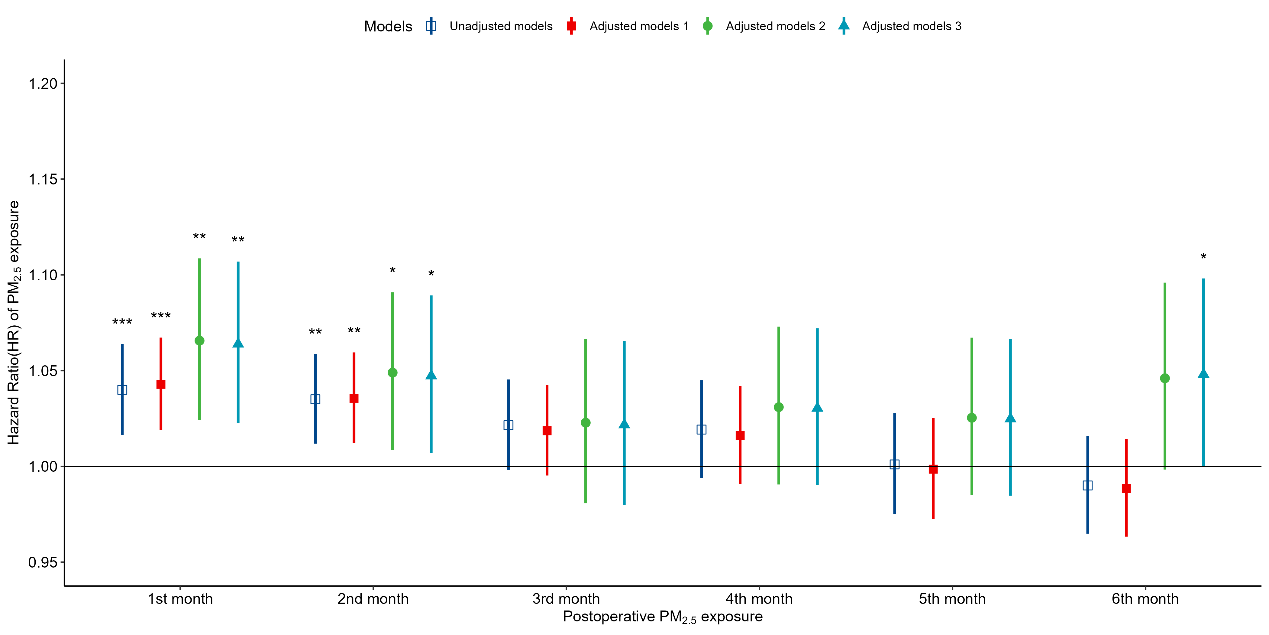


Figure S3 Hazard ratios (HRs) of postoperative death among lung cancer patients in association with specific monthly exposure to PM_2.5_, after adjusting for different age subgroups. Model 1 was adjusted for age at operation, sex, occupation type, ethnicity, marital status, and length of hospitalization. Model 2 was adjusted for the factors in Model 1 and operation season. Model 3 was adjusted for the factors in Model 2 and smoking and alcohol consumption. Age groups are <50, 50~59, 60~69, and ≥70.
